# Relapse-related long non-coding RNA signature to improve prognosis prediction of lung adenocarcinoma

**DOI:** 10.18632/oncotarget.8825

**Published:** 2016-04-18

**Authors:** Meng Zhou, Wanying Xu, Xiaolong Yue, Hengqiang Zhao, Zhenzhen Wang, Hongbo Shi, Liang Cheng, Jie Sun

**Affiliations:** ^1^ College of Bioinformatics Science and Technology, Harbin Medical University, Harbin 150081, PR China; ^2^ Medical Oncology Department, Affiliated Tumor Hospital, Harbin Medical University, Harbin 150001, PR China

**Keywords:** long non-coding RNA, lung adenocarcinoma, prognosis, relapse, recurrence-free survival

## Abstract

Increasing evidence has highlighted the important roles of dysregulated long non-coding RNA (lncRNA) expression in tumorigenesis, tumor progression and metastasis. However, lncRNA expression patterns and their prognostic value for tumor relapse in lung adenocarcinoma (LUAD) patients have not been systematically elucidated. In this study, we evaluated lncRNA expression profiles by repurposing the publicly available microarray expression profiles from a large cohort of LUAD patients and identified specific lncRNA signature closely associated with tumor relapse in LUAD from significantly altered lncRNAs using the weighted voting algorithm and cross-validation strategy, which was able to discriminate between relapsed and non-relapsed LUAD patients with sensitivity of 90.9% and specificity of 81.8%. From the discovery dataset, we developed a risk score model represented by the nine relapse-related lncRNAs for prognosis prediction, which classified patients into high-risk and low-risk subgroups with significantly different recurrence-free survival (HR=45.728, 95% CI=6.241-335.1; p=1.69e-04). The prognostic value of this relapse-related lncRNA signature was confirmed in the testing dataset and other two independent datasets. Multivariable Cox regression analysis and stratified analysis showed that the relapse-related lncRNA signature was independent of other clinical variables. Integrative in *silico* functional analysis suggested that these nine relapse-related lncRNAs revealed biological relevance to disease relapse, such as cell cycle, DNA repair and damage and cell death. Our study demonstrated that the relapse-related lncRNA signature may not only help to identify LUAD patients at high risk of relapse benefiting from adjuvant therapy but also could provide novel insights into the understanding of molecular mechanism of recurrent disease.

## INTRODUCTION

Lung cancer, including small cell lung cancer (SCC) and non-small cell lung cancer (NSCLC), is one of the most common cancers that severely threaten human health. The number of deaths from lung cancer is increasing, and it is estimated that nearly one in four cancer-related deaths is due to lung cancer [1]. Lung adenocarcinoma (LUAD) is the most frequent histological subtype of NSCLC and its incidence remains a rapidly increasing trend over the past few decades in China [2]. Despite improvement in diagnosis and treatment, the overall five-year survival rate for LUAD patients is only about 15% [3]. Moreover, more than 30% of patients treated with surgical resection will have a relapse within five years after surgery [4].

Recent advances in large-scale genomic analysis and high-throughput sequencing technologies have greatly increased our understanding of non-coding RNA (ncRNA) world. It has become increasingly apparent that a large proportion of human genome can be transcribed and produced a huge number of ncRNA molecules [5]. NcRNAs are briefly divided into two broad categories on the basis of their size: short ncRNAs and long ncRNAs. MicroRNA (miRNAs) is very important short ncRNAs, recently several predictors have been proposed to accurately predict miRNAs from other RNAs [6–8], which are very useful for the studies of the ncRNAs. Recently a web server called repRNA [9] was established to extract various features from RNA sequences, which will benefit the studies of RNAs. Long non-coding RNAs (lncRNAs), representing the largest class of ncRNAs, are mRNA-like transcripts and defined arbitrarily as ncRNAs of greater than 200 nucleotides in length [10, 11]. Evidence from growing publications has demonstrated that lncRNAs play important roles in various fundamental biological processes including development, differentiation and metabolism by executing functions as signals, decoys, guides and scaffolds [12, 13]. Furthermore, there is increasing evidence that lncRNAs are emerging as crucial components in the cancer paradigm [14]. The findings from transcriptome profiling analysis have shown highly aberrant lncRNA expression pattern in various types of human cancer [15, 16]. These differently expressed lncRNAs may be associated with tumorigenesis, tumor progression and metastasis [17, 18]. Moreover, lncRNA expression tended to be cell-, tissue- and cancer-type specific, thus making them attractive as independent biomarkers for diagnosis and prognosis [19]. Some well-known lncRNAs, such as *HOTAIR*, *MALAT1*, *H19*, *Xist*, *HULC* and *PTENP1*, have been found to possess oncogenic or/and tumor suppressor properties in various types of cancer [20, 21]. Several combinations of multiple lncRNAs were proposed as potential prognostic signature associated with overall survival in some cancers, including glioblastoma multiforme, colorectal cancer, breast cancer, oesophageal squamous cell carcinoma, non-small cell lung cancer and multiple myeloma [22–29]. Recent studies have shown the close relationship between cancer metastasis/relapse and dysregulated lncRNA expression [28, 30–34], implying the potential of lncRNAs as biomarkers to predict the risk of cancer metastasis/relapse. However, lncRNA expression patterns and their prognostic value for LUAD relapse have not been systematically elucidated.

In this study, we performed a systematic analysis of lncRNA expression profiles across 403 LUAD patients who did or did not relapse by repurposing the publicly available microarray expression profiles to determine whether there is significantly altered lncRNA expression pattern that could distinguish LUAD with relapse and without relapse. We aimed to detect potential lncRNA biomarkers closely correlated with LUAD relapse, and to develop novel lncRNA signature to identify LUAD patients who are at the higher risk for developing relapse.

## RESULTS

### Identification of altered lncRNA expression associated with tumor relapse

**Figure 1:**
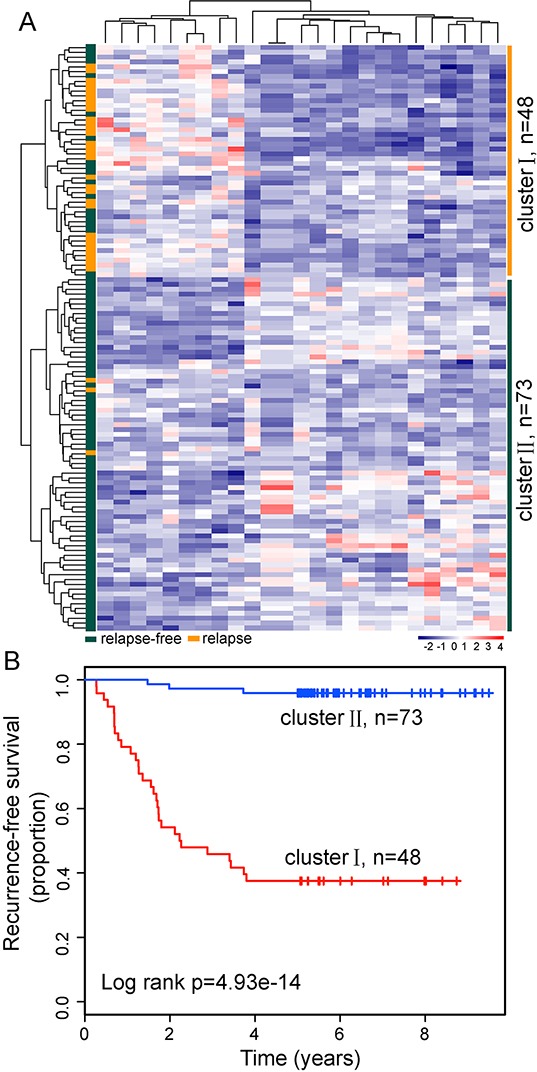
LncRNA expression patterns can distinguish patients who developed relapse from those who did not relapse in the discovery dataset **A.** The unsupervised hierarchical clustering heatmap of 121 patients based on the 25 differentially expressed lncRNAs in the discovery dataset. **B.** Kaplan-Meier survival curve for RFS in the two lncRNA transcriptomic classifications.

Here, the Okayama dataset, which is the largest dataset in our study, contains 64 LUAD patients who developed relapse and 162 relapse-free LUAD patients [35]. To identify relapse-related lncRNAs, we selected 88 favorable patients (alive > 5 years without any evidence of relapse) and 33 fatal samples (dead in 5 years with evidence of relapse) in the Okayama dataset to form a discovery dataset (n=121). The remaining patients in the Okayama dataset was considered as the internal testing dataset (n=105). Analysis of lncRNA expression profiles for LUAD patients in the discovery dataset revealed obvious differences and identified a total of 25 differentially expressed lncRNAs (adjusted p-value <0.01 after Bonferroni correction) between LUAD patients who developed relapse and relapse-free LUAD patients (Supplementary File S1). Unsupervised hierarchical clustering of 121 LUAD patients in the discovery dataset according to the expression patterns of these 25 differentially expressed lncRNAs showed two distant patient clusters, which were highly correlated with the tumor relapse status (p=7.557e-12, chi-square test; Figure 1A). Indeed, cluster I contained close to the majority of relapsed patients (n=30; 90.9%). Conversely, cluster II contained the majority of non-relapsed patients (n=70; 79.5%). Moreover, the Kaplan-Meier analysis and log-rank test revealed significant difference in recurrence-free survival (RFS) between these two patients subgroups (p=4.93-e14, log-rank test; Figure 1B). At three and five years, the RFS rates of LUAD patients in cluster II were 95.9% and 95.9%, respectively, whereas the corresponding rates in the cluster I were 45.8% and 37.5%, respectively. The above results demonstrated that dysregulated lncRNAs might have a predictive power in the prognosis of LUAD patients.

### Identification of optimal relapse-related lncRNA set

**Figure 2:**
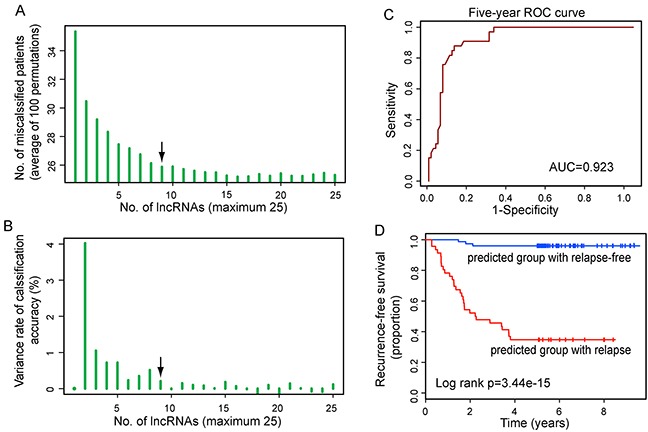
Identification of the relapse-related lncRNA signature in the discovery dataset **A.** The learning errors for top *N*-lncRNA model using 5-fold cross-validation procedures with 100 random partitions of the discovery dataset. **B.** The variance rate of classification accuracy when increasing numbers of the predictive lncRNAs. **C.** ROC analysis of the relapse-related lncRNA signature for relapse prediction within five years as the defining point. **D.** Kaplan-Meier survival curve for RFS of patients with relapse or relapse-free according to nine-lncRNA model-based prediction.

**Figure 3:**
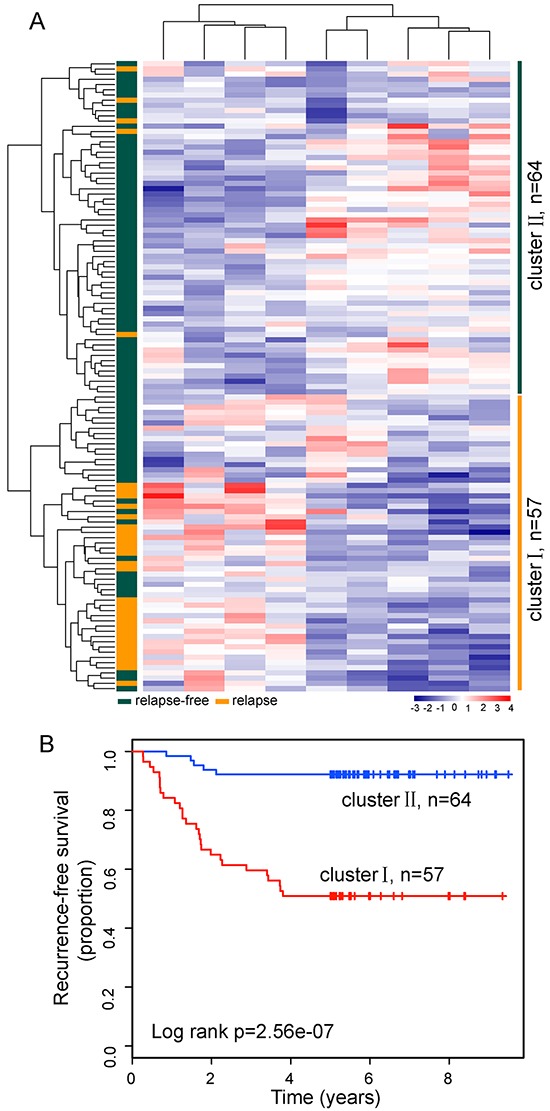
The heatmap and survival analysis of hierarchical clustering based on relapse-related lncRNA signature in the discovery dataset **A.** The unsupervised hierarchical clustering heatmap of 121 patients based on selected optimal nine lncRNAs in the discovery dataset. **B.** Kaplan-Meier survival curve for RFS in the two lncRNA transcriptomic classifications.

To identify relapse-related lncRNA signature, we used the weighted voting classification algorithm to predict outcome with the expression levels of these 25 differentially expressed lncRNAs as described in Materials and methods. These 25 differentially expressed lncRNAs were firstly ranked according to signal-to-noise metric. Then the average number of misclassified patients of the 5-fold cross-validation in 100 permutations was calculated when increasing numbers of top ranked predictive lncRNAs (Figure 2A). As a result, nine lncRNAs were found to yield a balance between accuracy and the number of lnRNAs, and were identified as optimal relapse-related lncRNA set. When choosing more than nine lncRNAs, there is a very slight increase in prediction accuracy (Figure 2B). With the selected nine lncRNAs and relapse status taken together, 121 LUAD patients were assigned as either relapse or relapse-free with accuracy of 84.3%. The classification of 121 LUAD patients produced a receiver operating characteristic (ROC) curve with AUC of 0.923, sensitivity of 90.9%, and specificity of 81.8% (Figure 2C). Furthermore, the Kaplan-Meier analysis for RFS demonstrated a significant difference between the groups predicted to be relapse or relapse-free (p=3.44e-15, log-rank test; Figure 2D). At three and five years, the RFS rates of LUAD patients in the predicted relapse-free group were 96% and 96%, respectively, whereas the corresponding rates in the predicted relapse group were 45.7% and 34.8%, respectively. We clustered LUAD patients in the discovery dataset according to the expression levels of nine relapse-related lncRNAs by hierarchical clustering analysis and obtained two distinctive patient groups with significantly different RFS (p=2.56e-07, log-rank test; Figure 3A and 3B). These results revealed better performance of nine relapse-related lncRNAs in prognosis prediction.

### Construction of relapse-related lncRNA signature from the discovery dataset

**Figure 4:**
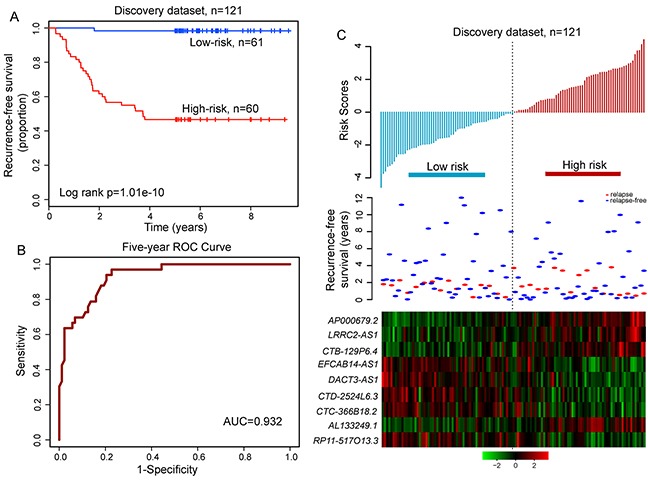
The relapse-related signature by risk score method in prognosis of RFS of patients with LUAD in the discovery dataset **A.** Kaplan-Meier survival curve for RFS of patients with high-risk or low-risk scores. **B.** ROC analysis of the risk score model for prognosis prediction within five years as the defining point. **C.** The distribution of risk scores, patients' relapse status and the heatmap of lncRNA expression profiles.

We applied univariate Cox proportional hazard regression to each of these nine relapse-related lncRNAs and found all of them significantly correlated with patient's RFS (Table 1). We then used these nine relapse-related lncRNAs to construct a signature by the risk score method as the classifier for prognosis prediction. This risk score model was defined as a linear combination of the expression levels of the nine relapse-related lncRNAs and the multivariate Cox regression coefficient as the weight as follows: (−1.049×expression value of *DACT3-AS1*) + (0.027×expression value of *CTD-2524L6.3*) + (0.485×expression value of *EFCAB14-AS1*) + (0.93×expression value of *AP000679.2*) + (0.439×expression value of *CTB-129P6.4*) + (0.091× expression value of *LRRC2-AS1*) + (−0.357×expression value of *RP11-517O13.3*) + (0.489×expression value of *AL133249.1*)+(0.015×expression value of *CTC-366B18.2*). Each LUAD patient in the discovery dataset was assigned a risk score and was classified into different prognostic groups (the high-risk group and low-risk group) according to the threshold of the median risk score (−0.054). The Kaplan-Meier analysis demonstrated a significant difference in RFS between two patient groups predicted to have good or poor prognosis (median RFS > 8 years vs. 3.73 years, p=1.01e-10, log-rank test; Figure 4A). The three-year and five-year RFS rates of the high-risk group were 55% and 46.7%, respectively, whereas the corresponding rates in the low-risk group were 98.4% and 98.4%, respectively. The univariate analysis revealed a significant association between the risk score and RFS, in which the hazard ratio (HR) of high-risk group versus low-risk group for RFS is 45.728 (95% confidence interval (CI) 6.241-335.1; p=1.69e-04; Table 2). The time-dependent ROC curves analysis for the relapse-related lncRNA signature prognostic model achieved an AUC of 0.932 at five years of RFS (Figure 4B). These results demonstrated that the relapse-related lncRNA signature has better performance in prognosis prediction of LUAD.

**Table 1:** Relapse-related lncRNAs significantly associated with RFS in patients with LUAD

Ensembl ID	Gene name	Chromosome	Coef	HR	95% CI of HR	*P*-value
ENSG00000245598	DACT3-AS1	Chr19: 46,660,364-46,677,447(+)	−0.964	0.382	0.26-0.559	7.52e-07
ENSG00000260037	CTD-2524L6.3	Chr15: 71,818,396-71,823,384(+)	−0.783	0.457	0.334-0.626	1.09e-06
ENSG00000228237	EFCAB14-AS1	Chr1: 46,674,036-46,692,098(+)	−1.226	0.294	0.182-0.474	5.13e-07
ENSG00000176984	AP000679.2	Chr11: 120,168,977-120,171,679(+)	1.058	2.881	1.976-4.202	3.83e-08
ENSG00000267282	CTB-129P6.4	Chr19: 44,882,027-44,890,876(−)	0.835	2.304	1.682-3.157	2.04e-07
ENSG00000268324	LRRC2-AS1	Chr3: 46,557,398-46,559,694 (+)	0.711	2.037	1.574-2.637	6.52e-08
ENSG00000258658	RP11-517O13.3	Chr14: 58,370,023-58,395,641(−)	−0.843	0.43	0.299-0.619	5.44e-06
ENSG00000223647	AL133249.1	Chr2: 31,793,823-31,803,980(−)	0.843	2.323	1.636-3.3	2.51e-06
ENSG00000248881	CTC-366B18.2	Chr5: 75,598,482-75,599,380(−)	−0.841	0.431	0.305-0.609	1.79e-06

Abbreviations: Coef, Coefficient; HR, hazard ratio; CI, confidence interval.

**Table 2:** Univariate and multivariate Cox regression analysis of RFS in each dataset

Variables		Univariate analysis			Multivariate analysis		
		HR	95% CI of HR	*P*-value	HR	95% CI of HR	*P*-value
**Discovery dataset, n=121**							
lncRNA signature	High risk vs. Low risk	45.728	6.241-335.1	1.69e-04	31.259	4.158-235.021	8.25e-04
Age	>65 vs. <=65	2.876	1.44-5.744	2.75e-03	2.771	1.314-5.845	0.007
Gender	Male vs. Female	1.623	0.818-3.222	0.166	1.23	0.505-2.995	0.649
Stage	II vs. I	4.761	2.397-9.455	8.31e-06	2.243	1.102-4.566	0.026
Smoking status	Yes vs. No	1.574	0.789-3.14	0.198	1.099	0.43-2.809	0.843
**Testing dataset, n=105**							
lncRNA signature	High risk vs. Low risk	11.02	3.346-36.3	7.96e-05	11.5	3.344-39.57	1.06e-04
Age	>65 vs. <=65	1.015	0.439-2.348	0.972	1.213	0.516-2.856	0.658
Gender	Male vs. Female	1.02	0.509-2.044	0.955	0.481	0.165-1.402	0.18
Stage	II vs. I	2.52	1.234-5.145	0.011	1.147	0.546-2.408	0.717
Smoking status	Yes vs. No	1.197	0.597-2.399	0.612	1.695	0.585-4.913	0.331
**Okayama dataset, n=226**							
lncRNA signature	High risk vs. Low risk	20.66	7.5-56.91	4.7e-09	17.619	6.266-49.543	5.36e-08
Age	>65 vs. <=65	1.801	1.72-3.025	0.026	1.949	1.145-3.315	0.014
Gender	Male vs. Female	1.268	0.783-2.055	0.335	0.905	0.471-1.738	0.765
Stage	II vs. I	3.37	2.066-5.497	1.13e-06	1.609	0.969-2.67	0.066
Smoking status	Yes vs. No	1.324	0.816-2.148	0.256	1.09	0.565-2.102	0.797
**Der dataset, n=124**							
lncRNA signature	High risk vs. Low risk	1.829	1.074-3.115	0.026	1.938	1.135-3.31	0.015
Age	>65 vs. <=65	1.327	0.756-2.328	0.324	1.397	0.788-2.477	0.252
Gender	Male vs. Female	1.267	0.761-2.109	0.364	1.312	0.778-2.213	0.308
Stage	II vs. I	2.64	1.559-4.469	3.02e-04	2.746	1.615-4.67	1.92e-04
Smoking status	Yes vs. No	1.219	0.628-2.366	0.559	0.968	0.49-1.914	0.926
**Botling dataset, n=53**							
lncRNA signature	High risk vs. Low risk	ç1.86	0.939-3.685	0.075	1.997	0.976-4.084	0.058
Age	>65 vs. <=65	1.231	0.653-2.321	0.521	1.118	0.58-2.155	0.74
Gender	Male vs. Female	1.123	0.59-2.138	0.725	1.152	0.552-2.404	0.706
Stage	I	1.000 (reference)			1.000 (reference)		
	II	1.823	0.796-4.176	0.156	2.074	0.878-4.898	0.096
	III	2.338	1.027-5.322	0.043	2.624	1.066-6.459	0.036
	IV	4.61	0.588-36.143	0.146	3.866	0.46-32.479	0.213

Abbreviations: HR, hazard ratio; CI, confidence interval.

The distribution of prognostic risk scores, the relapse status and expression pattern of lncRNA signature of 121 LUAD patients in the discovery dataset was shown in Figure 4C. Of these nine relapse-related lncRNAs, five were protective lncRNAs whose high expression were associated with low risk, and the remaining four were risky lncRNAs whose high expression were associated with high risk.

### Validation of relapse-related lncRNA signature in the testing and entire okayama dataset

**Figure 5:**
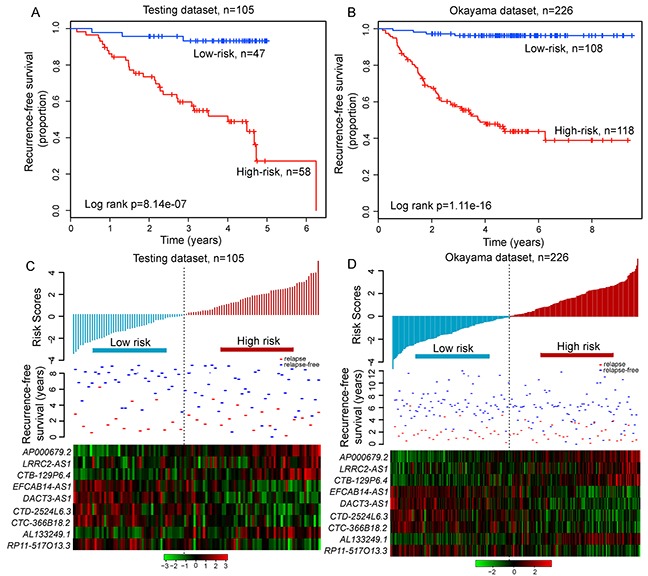
The relapse-related signature predicts RFS of patients with LUAD in the testing dataset and entire Okayama dataset Kaplan-Meier survival curves of RFS between high-risk and low-risk patients in **A.** the testing dataset and **B.** entire Okayama dataset. The distribution of risk scores, patients' relapse status and the heatmap of lncRNA expression profiles in **C.** the testing dataset and **D.** entire Okayama dataset.

To confirm our findings, the predictive ability of relapse-related lncRNA signature was validated in LUAD patients from the testing dataset and entire Okayama dataset. With the same risk score formula and cutoff value derived from the discovery dataset, patients of the testing dataset were classified into the high-risk group (n=58) and low-risk group (n=47). As in the discovery dataset, the RFS time of patients in the high-risk group was significantly shorter than that in the low-risk group (median RFS 4.01 years vs. > 5 years, p=8.14e-07, log-rank test) (Figure 5A). The risk stratification of the entire Okayama dataset (i.e. combined the discovery and testing series) also yielded similar result. This relapse-related lncRNA signature was able to separate 226 patients in the entire Okayama dataset into two groups with significantly different RFS (median 3.73 years vs. > 8 years, p=1.11e-16, log-rank test) (Figure 5B). A significant association between the relapse-related lncRNA signature and RFS in the univariate Cox regression analysis was observed both in the testing and entire Okayama datasets. The hazard ratios of the high-risk group versus the low-risk group for RFS was 11.02 (p=7.96e-05; 95% CI 3.346-36.3) in the testing dataset, and 20.66 (p=4.7e-09; 95% CI 7.5-56.91) in the entire Okayama dataset (Table 2). The distribution of prognostic risk scores, the relapse status and expression pattern of lncRNA signature of LUAD patients in the testing and entire Okayama datasets were shown in Figure 5C and 5D. Patients with high prognostic scores tended to express risky lncRNAs, whereas patients with low prognostic scores tended to express protective lncRNAs.

### Further validation of relapse-related lncRNA signature with two additional independent datasets of LUAD patients

**Figure 6:**
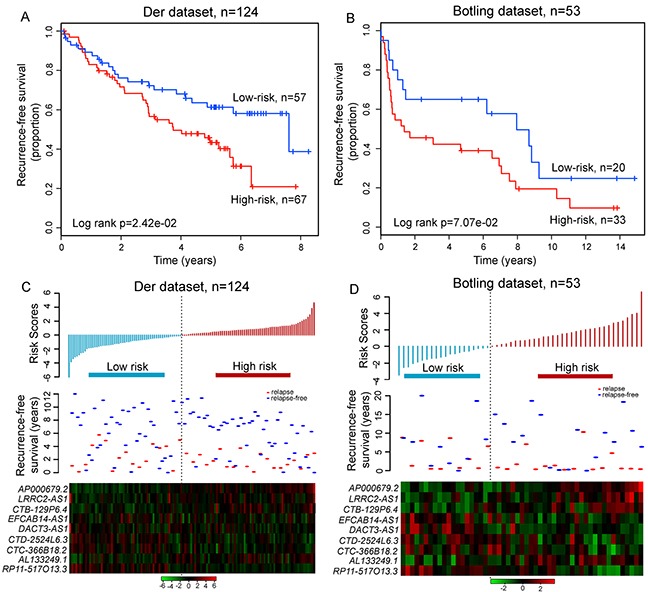
Independent validation of relapse-related signature for prognosis prediction in two additional independent datasets Kaplan-Meier survival curves of RFS between high-risk and low-risk patients in **A.** the Der dataset and **B.** the Botling dataset. The distribution of risk scores, patients' relapse status and the heatmap of lncRNA expression profiles in **C.** the Der dataset and **D.** the Botling dataset.

Further validation of the prognostic power of relapse-related lncRNA signature in LUAD patients was conducted using two additional completely independent cohorts of 124 and 53 LUAD patients obtained from Der's study [36] and Botling's study [37], which will be further referred to as the Der dataset and Botling dataset. The median cutoff value of risk score obtained from the discovery dataset was used for the Der dataset and Botling dataset to classify patients into either high-risk or low-risk groups. For the Der dataset, patients with high-risk scores had significantly shorter RFS than those with low-risk scores (median 3.74 years vs. 7.61 years, p=2.42e-02, log-rank test) (Figure 6A). Among patients in the Botling dataset, the high-risk group and low-risk group were marginally significantly different in their RFS (median 1.38 years vs. 7.96 years, p=7.07e-02, log-rank test) (Figure 6B). At three and five years, the respective absolute differences in RFS between the high-risk group and low-risk group were 15.6% (56.6% vs.72.2%) and 18% (43.3% vs. 61.3%) for Der dataset, and 22.8% (42.2% vs. 65%) and 26.1% (38.9% vs. 65%) for Botling dataset, respectively. In the univariate analysis, the hazard ratios of high-risk scores versus low-risk scores for RFS were 1.829 (p=2.62e-02; 95% CI 1.074-3.115) for Der dataset and 1.86 (p=7.52e-02; 95% CI 0.939-3.685) for Botling dataset, respectively (Table 2). The distribution of prognostic risk scores, the relapse status and expression pattern of lncRNA signature of LUAD patients in the two independent cohorts were consistent with those observed in the discovery, testing and entire Okayama datasets (Figure 6C and 6D).

### Independence of prognostic value of relapse-related lncRNA signature from other clinical variables

**Figure 7:**
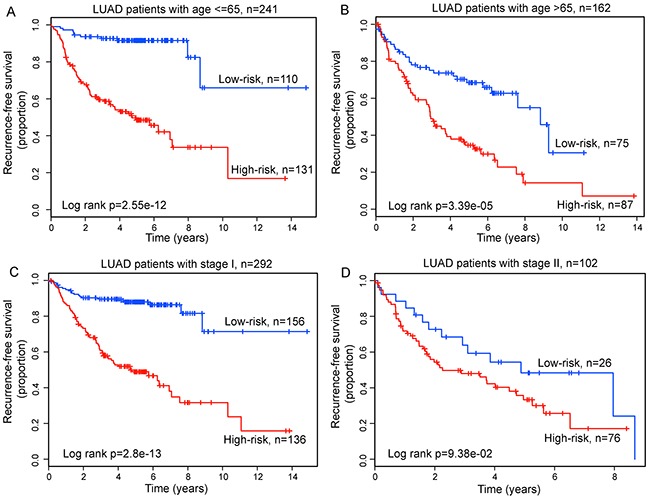
Prognosis prediction in patients stratified by age and tumor stage Kaplan-Meier survival curves for younger patients **A.** and elder patients **B.** Kaplan-Meier survival curves for stage I patients **C.** and stage II patients **D.**

To determine whether the prognostic value of the relapse-related lncRNA signature was independent of other clinical variables, we conducted a multivariate Cox regression analysis including lncRNA signature, age, gender, smoking status and tumor stage as covariates. The results of multivariable Cox regression analysis from five LUAD patient datasets showed that the relapse-related lncRNA signature was still significantly associated with RFS after adjusted by these clinical variables in each dataset (Table 2). We also found that age and tumor stage was significant in the multivariate analysis in some datasets. So we performed data stratification analysis according to the age and tumor stage. All LUAD patients enrolled in this study were first stratified into either the younger stratum (age≤65) or the elder stratum (age>65). This analysis showed that within each age stratum, the relapse-related lncRNA signature could further classified patients into the high-risk group and low-risk group with significantly different RFS (median 4.73 years vs. > 8.68 years, p=2.55e-12 for younger stratum; median 2.94 years vs. 8.83 years, p=3.39e-05 for elder stratum; log-rank test) (Figure 7A and 7B). Next, the stratified analysis was carried out in tumor stage, which stratified patients into the stage I stratum and stage II stratum. For patients within stage I stratum, significant differences for RFS between high-risk group and low-risk group were observed (median 4.68 years vs. >10 years, p=2.8e-13, log-rank test) (Figure 7C). Among stage II patients, RFS was also marginally significantly different between the groups with high-risk and low-risk scores (median 2.26 years vs. 4.88 years, p=9.38e-02, log-rank test) (Figure 7D). Because of limited patient size, the stratified analysis was not conducted for stage III (n=8) and IV (n=1) patients. Taken together, the results of multivariate Cox regression and stratification analyses suggested that the relapse-related lncRNA signature is independent of other clinical features for prognosis prediction of LUAD patients. Moreover, the discrimination performance of relapse-related lncRNA signature measured by the C-index was much higher than that of other clinical variables in each dataset (Table 3), demonstrating the better predictive ability to discriminate between LUAD patients who are or not likely to develop relapse.

**Table 3:** Concordance index values of the relapse-related lncRNA signature and other clinical feature for prognosis prediction

	C-index				
Variables	Discovery dataset	Testing dataset	Okayama dataset	Der dataset	Botling dataset
lncRNA signature	0.876	0.761	0.822	0.677	0.657
Age	0.608	0.498	0.556	0.528	0.514
Gender	0.567	0.490	0.536	0.536	0.534
Stage	0.671	0.590	0.635	0.591	0.585
Smoking	0.561	0.525	0.544	0.531	-

“-” means no corresponding information available in this dataset.

### Identification of associated biological functions of relapse-related lncRNA signature

**Figure 8:**
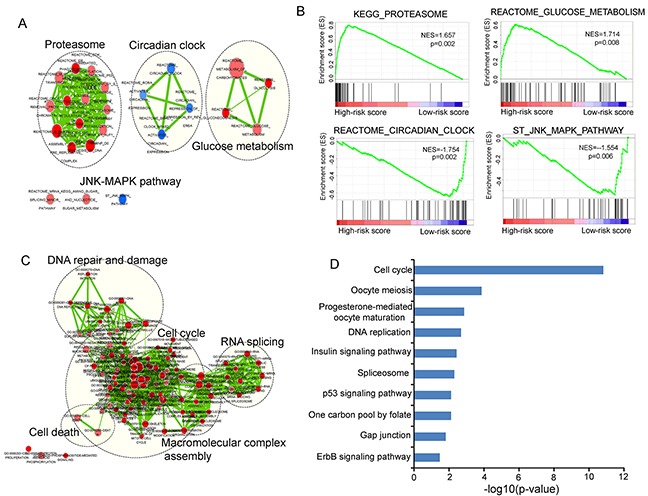
Functional analysis of the prognostic lncRNAs **A.** The enrichment map of gene sets with each node represents a gene set and an edge represents the proportion of shared genes between connecting gene sets. **B.** The enriched biological pathways and processes associated with risk score. **C.** The functional enrichment map of GO terms with each node represents a GO term and an edge represents the proportion of shared genes between connecting GO terms. **D.** The enriched KEGG pathways ranked by −log_10_ (p-value).

As an initial step toward gaining insights into the biological functions of relapse-related lncRNA signature, we first applied GSEA to identify associated biological pathways and processes from gene expression profiles of LUAD patients in the high-risk and low-risk groups classified by the relapse-related lncRNA signature in the Okayama dataset. The high-risk scores were associated with coordinated transcriptional up-regulation of multiple gene sets (Figure 8A and 8B) (Supplementary File S2), mainly involved in glucose metabolism and proteasome that have been reported to be involved in lung cancer [38, 39]. The low-risk score was accompanied with up-regulation of circadian clock and JNK-MAPK pathway (Figure 8A and 8B) (Supplementary File S2), both of which have inhibitory effects on the growth of lung cancer [40, 41]. Then we measured the co-expressed relationships between nine relapse-related lncRNAs and mRNAs by calculating the Pearson correlation coefficient of paired lncRNA and mRNA expression profiles and identified 1414 mRNAs positively correlated (ranked top 1%) with at least one of nine relapse-related lncRNAs. The functional enrichment analysis of GO and KEGG pathway revealed that the co-expressed mRNAs were most significantly enriched in 15 GO functional annotation clusters (mainly involved in cell cycle, DNA repair and damage, macromolecular complex assembly, RNA splicing and cell death) (Figure 8C) (Supplementary File S3), and 10 KEGG pathways including cell cycle, oocyte meiosis, progesterone-mediated oocyte maturation, DNA replication, insulin signaling pathway, spliceosome, p53 signaling pathway, One carbon pool by folate, gap junction and ErbB signaling pathway (p<0.05 and Fold Enrichment>2) (Figure 8D) (Supplementary File S4). This integrative functional analysis suggested that the dysregulated expression of relapse-related lncRNAs might affect the critical biological pathways and processes involved in tumor progression and recurrence.

## DISCUSSION

LUAD, the most frequent type of NSCLC, remains to be the leading cause of cancer-related deaths in women and men. LUAD is a recurrent disease, and more than 30% of patients still faced relapse after surgical resection and treatment and ultimately die of relapse [4]. In the past years, great efforts have been made to improve our understanding of the possible molecular mechanism of relapse process at protein, mRNA and microRNA (miRNA) levels, and some protein/mRNA/miRNA-based predictive signature were reported to identify patients at the high risk of relapse, which will enable them to benefit from adjuvant therapy [35, 42–50]. LncRNAs is a novel layer of gene regulation network and their aberrant expression has been demonstrated to be associated with tumorigenesis, tumor progression and metastasis [14, 17, 18, 30]. Until now, several lncRNAs, including *MALAT-1*, *CCAT2*, *HOTAIR*, and *ZXF1*, have been found to contribute to LUAD [51–54]. More recently, differentially expressed lncRNAs were observed between LUAD tissues and normal tissues by microarray analysis [55]. However, lncRNA expression patterns and their prognostic value for LUAD relapse have not been systematically investigated.

In the present study, we obtained lncRNA expression profiles in 403 LUAD patients by repurposing the publicly available microarray expression profiles, and performed comparison analysis between the groups of 121 LUAD patients that dead in 5 years with evidence of relapse and those that alive > 5 years without any evidence of relapse. Although Okayama *et al.* analyzed ALK-positive and triple negative LUAD patients [35], current study did not differentiate them. We found that two patient groups have significantly different lncRNA expression patterns and identified 25 differentially expressed lncRNAs. Hierarchical clustering analysis revealed that these differentially expressed lncRNAs were significantly correlated with LUAD relapse. By using the lncRNA expression data and a weighted voting algorithm, we were able to generate an optimal set of nine lncRNAs that could clearly distinguish patients who developed relapse from those who did not relapse with high accuracy, demonstrating their potential clinically application to identify patients with higher risk of relapse and improve prognosis prediction of LUAD. In statistical prediction, the following three cross-validation methods are often used to examine a predictor for its effectiveness in practical application: independent dataset test, subsampling test, and jackknife test. However, of the three test methods, the jackknife test is deemed the least arbitrary that can always yield a unique result for a given benchmark dataset as elaborated in [56]. Accordingly, the jackknife test has been widely recognized and increasingly used by investigators to examine the quality of various predictors [57–61]. However, to reduce the computational time, we adopted the 5-fold cross-validation in this study as done by many investigators with SVM as the prediction engine. Furthermore, the small subset of predictive lncRNAs perhaps not only has better transferability in the clinics but also reduce the possibilities of false positives in the selection of predictive lncRNAs. By focusing on relapse-related lncRNAs, we constructed a nine-lncRNA signature for prognosis by the risk score method based on the linear combination of expression data of relapse-related lncRNAs and weighted by the regression coefficients from multivariate Cox regression analysis, which effectively classified patients of the discovery dataset into high-risk group and low-risk group with significantly different RFS. Moreover, the prognostic power of this relapse-related lncRNA signature was further validated by the testing dataset and other two independent non-overlapping datasets, indicating good reproducibility and robustness of relapse-related lncRNA signature in patient prognosis.

The conventional indicators for making adjuvant treatment decisions for patients after surgical resection are based on some clinical factors, such as tumor stage, tumor size, margin status and so on [45]. Therefore, we performed multivariate Cox regression analysis to assess the independence of the relapse-related lncRNA signature in prognosis prediction, and in these datasets, the signature maintained an independent correlation with RFS after adjusting for age, gender, stage and smoking status (Table 2). However, it can be found that age and stage also were two important factors affecting RFS when accessed in the multivariate Cox regression analysis. So, we further stratified patients according age and stage, and applied this lncRNA signature to classify patients within the same age stratum or the same stage into two subgroups with good and poor prognosis. The stratification analysis showed that patients in the predicted good prognosis group tended to have longer RFS than those in the predicted poor prognosis group across these stratified patient datasets, demonstrating the age- and stage-independent prognostic value of this lncRNA signature. Moreover, the discriminatory power of relapse-related lncRNA signature measured by the C-index value was better than the discrimination provided by the clinical variables in different datasets. Taken together, this relapse-related lncRNA signature was a significant and independent prognostic marker in predicting relapse risk of patients with LUAD.

Although the number of identified lncRNAs in human is continuously increasing, only a small proportion have been well functionally characterized to date and the functional study of lncRNAs remains to be in its infancy. For example, only 181 lncRNAs with functional evidence were recorded in lncRNAdb v2.0 database by manually literature mining [62]. To gain functional insight into these nine relapse-related lncRNAs, we performed an integrative bioinformatics analysis to predict lncRNA function which could overcome the bias derived from the single prediction method. We found that the GO composition of co-expressed mRNAs with these lncRNAs revealed biological relevance to disease relapse, such as cell cycle, DNA repair and damage and cell death. Moreover, the pathway analysis also revealed a significant enrichment of co-expressed mRNAs with these lncRNAs in lung cancer-related pathways. These in silico functional analysis based on the co-expressed mRNAs with lncRNAs suggested that expression variation of these relapse-related lncRNAs might affect some critical biological pathways and processes involved in tumor progression and recurrence. However, biological significance of these relapse-related lncRNAs should be validated using wet experiments on cell lines and clinical samples in the future. As shown in a series of recent publications [63–68] in developing or reporting new methods or findings, user-friendly and publicly accessible web-servers will significantly enhance their impacts [69], we shall make efforts in our future work to provide a web-server for the method presented in this article.

In summary, we investigated lncRNA expression patterns in LUAD relapse and their effects on patient outcome for the first time. We identified an optimal small set of nine lncRNAs whose expression patterns were able to discriminate between relapsed and non-relapsed LUAD patients with sensitivity of 90.9% and specificity of 81.8%. A relapse-related lncRNA signature was developed by the risk model method that effectively classified patients into good and poor prognosis groups across different datasets. Moreover, the prognostic power of this signature was independent of other clinical variables. The relapse-related lncRNA signature may not only help to identify LUAD patients at high risk of relapse benefiting from adjuvant therapy but also could provide novel insights into the molecular mechanism of recurrent disease.

## MATERIALS AND METHODS

### LUAD patient datasets

LUAD patients with whole-genome gene expression profiles (generated from the Affymetrix Human Genome U133 Plus 2.0 Array) and corresponding clinical information were collected from the publicly available Gene Expression Omnibus (GEO) database (http://www.ncbi.nlm.nih.gov/geo/). A total of 403 LUAD patients were enrolled in this study, including 226 patients from Okayama's study (the accession number is GSE31210, http://www.ncbi.nlm.nih.gov/geo/query/acc.cgi?acc=GSE31210) [35], 124 patients from Der's study (the accession number is GSE50081, http://www.ncbi.nlm.nih.gov/geo/query/acc.cgi?acc=GSE50081) [36] and 53 patients from Botling's study (the accession number is GSE37745, http://www.ncbi.nlm.nih.gov/geo/query/acc.cgi?acc=GSE37745) [37]. LUAD patients and tumor features are detailed in Table 4.

**Table 4:** Clinical features of LUAD patients enrolled in this study

Covariates		Discovery dataset N=121	Testing dataset N=105	Okayama dataset N=226	Der dataset N=124	Botling dataset N=53
Age (years), no (%)	<=65	93(76.9)	83(79.0)	176(77.9)	40(32.3)	25(47.2)
	>65	28(23.1)	22(21.0)	50(22.1)	84(67.7)	28(52.8)
Gender, no (%)	Male	55(45.6)	50(47.6)	105(46.5)	63(50.8)	20(37.7)
	Female	66(54.4)	55(52.4)	121(53.5)	61(49.2)	33(62.3)
Vital status, no (%)	Alive	88(72.7)	103(98.1)	191(84.5)	75(60.5)	16(30.2)
	Dead	33(27.3)	2(1.90)	35(15.5)	49(39.5)	37(69.8)
Relapse status, no (%)	Relapse	33(27.3)	31(29.5)	64(28.3)	37(29.8)	26(49.1)
	Not relapse	88(72.7)	74(70.5)	162(71.7)	87(70.2)	27(50.9)
Tumor stage, no (%)	I	93(76.9)	75(71.4)	168(74.3)	90(72.6)	34(64.2)
	II	28(23.1)	30(28.6)	58(25.7)	34(27.4)	10(18.9)
	III	-	-	-	-	8(15.1)
	IV	-	-	-	-	1(1.8)
Smoking status, no (%)	Never-smoker	62(51.2)	53(50.5)	115(50.9)	23(18.5)	-
	Ever-smoker	59(48.8)	52(49.5)	111(49.1)	90(72.6)	-
	Undetermined	-	-	-	11(8.9)	-

“-” means no corresponding information available in the dataset.

### Acquisition and analysis of lncRNA expression profiles of LUAD patients

The raw array data (.CEL files) of 403 LUAD patients were retrieved from the GEO database and were uniformly pre-processed using the Robust Multichip Average (RMA) algorithm for background correction, quantile normalization and log2-transformation [70]. To account for the heterogeneity of multiple microarray datasets in systematic measurement, each dataset was standardized independently by the Z-score transformation to scale expression intensities of each probe as follows [71]:

$$\text{Z score }=(e_{i}-\bar{e})/\delta$$

Where *e_i_* the raw intensity data of probe*i*, $\bar{e}$ is the overall average intensity of probes in a single experiment and δ is the standard deviation (SD) of all of the measured intensities.

The probe sequences of Affymetrix HG-U133 Plus 2.0 array were obtained from the Affymetrix website (http://www.affymetrix.com). LncRNA expression data of 403 LUAD patients were obtained by repurposing Affymetrix array probes as previous described [72, 73]. Briefly, probe sets for Affymetrix HG-U133 Plus 2.0 array were re-annotated to the human genome (GRCh38) and lncRNA genes based on the annotations from GENCODE (release 21) using SeqMap tool [74]. Then those probes (or probe sets) that were uniquely mapped to the human genome and lncRNA genes with no mismatch were generated to represent the lncRNAs. For each lncRNA, all corresponding probe set signals were averaged to produce a single expression value. Finally, the expression data of 2313 lncRNAs was obtained.

The expression profiles of lncRNAs between relapse-free LUAD patients (alive > 5 years without any evidence of relapse) and LUAD patients who developed relapse (dead in 5 years with evidence of relapse) were compared and the differentially expressed lncRNAs were identified using two-tailed T-test. Those lncRNAs with an adjusted p-value <0.01 after Bonferroni correction were considered as differentially expressed lncRNAs. The unsupervised hierarchical clustering of both LUAD patients and lncRNAs was performed with R software using the euclidean distance and complete linkage method.

### Identification of relapse-related lncRNA set

To identify optimal lncRNA set associated with relapse of LUAD patients, we used the weighted voting algorithm to develop a supervised classification model, assessed using 5-fold cross-validation with 100 randomized permutations in the discovery dataset as follows: (i) patients of discovery dataset were divided into five non-overlapping sets with equal quantity. (ii) With four of five sample sets, the signal-to-noise statistic (*S*_lnc-*i*_) of each lncRNA was calculated as

$$s_{\text{Inc}-i}=\left(\mu_{\text{relapse}}\;(\text{In}c-i)-\mu_{\text{non-relapse}}(\text{In }c-i)\right)/\left(\sigma_{\text{relapse}}(\text{In }c-i)+\sigma_{\text{non-relapse}}(\text{In }c-i)\right),$$

where $\mu_{\text{relapse}}(\text{In }c-i)$ and $\sigma_{\text{relapse}}(\text{In }c-i)$ $\left(\mu_{\text{non-relapse}}(\text{In }c-i\right)\text{ and }\sigma_{\text{nonrelapse}}(\text{In }c-i))$ is the mean value and standard deviation (SD) of expression level of lncRNA *i* in LUAD patients who developed relapse (relapse-free). In addition, the classification boundary of two classes (relapse or non-relapse) for each lncRNA *i* was also calculated as

$$b_{\text{In }c-i}=\left(\mu_{\text{relapse}}(\text{In }c-i)+\mu_{\text{non-relapse}}(\text{In }c-i)\right)/2$$

(iii) lncRNAs were ranked according to its signal-to-noise statistic with absolute value, and top *N* ranked lncRNA were selected to develop a supervised classification model. The top *N* was initially set to top 1, and increase one lncRNA each time until *N* was equal to the number of candidate lncRNAs. (iv) The above classification model was used to classify patients in the remaining one set into relapse or non-relapse based on voting rules: each lncRNA *i* in the top *N* model has a vote *V*_ln c−*i*_ ($V_{\text{In }c-i}(V_{\text{In }c-i}=S_{\text{In }c-i}(e_{\text{In }c-i}-b_{\text{In }c-i})$, where *e*_ln c–*i*_ is the expression level of lncRNA *i* in the corresponding patient. The votes of top *N* ranked lncRNAs were summed to determine the relapse or relapse-free class of sample. (v) repeat steps i-iii for each of the five non-overlapping sets. (vi) The 5-fold cross-validation process was repeated 100 times. The average number of misclassified patients of 100 randomized permutations for top *N* model as follows:

$$\text{averag}e_{\text{errorN}}=(\sum_{i=1}^{100}\sum_{j=1}^{5}\text{error})/100.$$

Finally, the top *N* which yielded the optimal numbers of learning errors was selected as the optimal number (OPN) of predictive lncRNAs. The frequencies of lncRNAs in 500 candidate lncRNAs ranking list according to their signal-to-noise statistic were ranked and top OPN of lncRNAs were identified as optimal relapse-related lncRNA signature.

### Statistical analysis for classification and prediction

The association between expression levels of relapse-related lncRNAs and patients' RFS was assessed using the univariate Cox regression analysis. RFS was calculated as the time to tumor recurrence or death due to any cause, and was censored at the time of last following-up when recurrence has happened. To construct a prognostic model, the relapse-related lncRNAs were fitted in the multivariate Cox regression model in the discovery dataset. Then we applied these relapse-related lncRNAs to build an expression signature by risk score method as follows [75]:

$$\text{Risk Score}=\sum_{i=1}^{N}\left(\text{Ex}p_{i}*w_{i}\right)$$

Where *N* is the number of relapse-related lncRNAs, *Exp_i_* is the expression levels of ln*cRNA_i_*, and *w_i_* is the estimated regression coefficient of ln*cRNA_i_* derived from the above multivariable Cox regression analysis in the discovery dataset. This relapse-related lncRNA expression signature was established by taking into account the contribution of independent relapse-related lncRNA to patient's RFS. Finally, LUAD patients were assigned a risk score according to the relapse-related lncRNA expression signature, and were divided into high-risk and low-risk groups using the median of the risk score generated from the discovery dataset as the cutoff value. The LUAD patients with higher scores were considered to have high risk of poor outcome. The difference in RFS between high-risk group and low-risk group was demonstrated by Kaplan-Meier survival plots, and the statistical significance was assessed by two-sided log-rank test. Univariate and multivariate analyses with Cox proportional hazards regression were performed with RFS as the dependent variable and relapse-related lncRNA risk score and clinical features as explanatory variables in each dataset. Hazard ratio (HR) and 95% confidence intervals (CI) was estimated by Cox proportional hazards regression model. The sensitivity and specificity of relapse-related lncRNA risk score in RFS prediction was evaluated by analysis of the time-dependent ROC curve within 5 years as the defining point. The Harrell's concordance index (C-index) was calculated to quantify the discriminatory power of relapse-related lncRNA risk score [76]. A C-index of 1.0 indicates perfect prediction accuracy, whereas a C-index of 0.5 represents prognosis prediction is equivalent to random guessing. All statistical analyses were performed using R software and Bio-conductor.

### Integrative prediction analysis of lncRNA function

In order to explore the potential biological roles of lncRNA, we performed function enrichment analysis by the integration of gene sets, Gene Ontology (GO) and Kyoto encyclopedia of genes and genomes (KEGG). Gene set enrichment analysis (GSEA) was performed by the JAVA program using MSigDB (c2.cp.v5.0, 1330 gene sets) to rank gene set associated with risk score by enrichment score [77]. The gene sets with positive enrichment score (or negative enrichment score) and p-value <0.01 after performing 1000 permutations of the risk-phenotype labels were considered as significant enriched gene sets in which most of the genes are up-regulated accompanied with high-risk scores (or low-risk scores). GO and KEGG enrichment analysis were carried out to assess over-representation of functional categories among a gene set of interest using DAVID Bioinformatics Tool (version 6.7) limited to GO terms in the “Biological Process”(GOTERM-BP-FAT) and KEGG pathway categories [78]. Functional categories with p-value of <0.05 and an enrichment score of >2 using the whole human genome as background were considered significant. Significant functional annotations of GSEA and GO analysis were organized into an interaction network with similar functions using the Enrichment Map [79] plugin in Cytoscape 3.2.1 [80].

## SUPPLEMENTARY FILES










