# Supplementary material for: Relapse-related long non-coding RNA signature to improve prognosis prediction of lung adenocarcinoma
Source: Oncotarget. 2016 Apr 18;7(20):29720–38. doi: 10.18632/oncotarget.8825 (PMC5045428; doi:10.18632/oncotarget.8825)
Supplement: Supplementary file 1 [file oncotarget-07-29720-s001.pdf]

SUPPLEMENTARY FILES

Supplementary File S1: The differentially expressed lncRNAs between relapse-free LUAD patients (alive > 5 years) and LUAD patients who developed relapse (dead in 5 years) using two-tailed T-test with an adjusted p-value <0.01 after Bonferroni correction.

See Supplementary File 1

Supplementary File S2: Gene set enrichment results from gene expression profiles of LUAD patients with high-risk and low-risk group classified by the relapse-related lncRNA signature.

See Supplementary File 2

Supplementary File S3: Functional annotation clusters of enriched GO terms.

See Supplementary File 3

Supplementary File S4: KGEE pathway enrichment results.

See Supplementary File 4
